# Progress in Otology

**Published:** 1895-11-16

**Authors:** 


### PROGRESS IN OTOLOGY.

**Etiology of Deafness.**—W. W. Ireland[1] states that it has been found that there is a much larger number of deaf persons in districts where goitre and cretinism prevail. Deafness is one of the manifestations of the cretino-genetic miasma. Sir Henry Barkly has reported that deafness is more than twice as common among the population of European descent in the Cape Colony than it is in England, namely, one in 809 to one in 1,640. Among the Kaffirs it is but one in 2,820. Dr. Holger Mygind shows in a convincing manner the hereditary character of deafness, in his work on deaf mutism. In every thirtieth or thirty-first marriage in which but one parent was deaf there was a deaf child, but where both parents were deaf there was a deaf mute out of every sixth marriage. From Hartmann's statistics we gather that every fifty-seventh or fifty-eighth child born in marriage contracted by deaf persons inherits the deficiency. Mygge finds in the marriages of deaf parents, including those in which both or only one parent is deaf, that every sixty-second child is so affected, which is certainly much greater than the proportion in the general population. From other sources the fact has been elicited that the probability of congenital deafness is nearly seven times greater when both parents are deaf than when only one is so. Another interesting statement, the outcome of observations by Turner, of the Columbia Institution, is that one-tenth of the children born of parents of whom one was a deaf mute, were afflicted in the same manner, one-third of the children being deaf mutes when both parents were the subjects of that condition. Thus the proportion of deaf children when both parents are so affected is more than three times greater than when only one parent is; and we may conclude that congenital deaf mutism is the result of hereditary influences, which are intensified when coming from both parents. There are often several deaf children in a family: it has been found that about 40 per cent. of congenital deaf mutes had deaf and dumb brothers. With reference to this point, viz., the relationship of the deaf, Mygind says that deafness is comparatively frequent among the relatives of deaf mutes. It is least frequent in the direct ascending line (parents, grandparents), more so in the collateral branches (uncles, aunts), but most of all amongst the brothers and sisters, and therefore one is justified in supposing that the manner in which deaf mutism appears in different generations is a result of certain qualities appertaining to its congenital form. Consanguineous marriages are believed to play a part in favouring congenital deafness. Tuberculosis and drunkenness also appear as causes; about 11 per cent. of the parents of deaf mutes were found by Mygge to be addicted to drink, and 8·7 of the parents of children suffering from acquired deafness. It is a curious fact that illegitimate children are less liable to either idiocy or deafness than those born in wedlock.

**Oral Instruction of the Deaf.**—J. D. Wright,[2] in a letter to the "Medical Record," informs us as to the progress that is being made in this method of teaching the deaf, and it is truly marvellous to contemplate the state of perfection to which it has been brought. Great results are being obtained by acoustic gymnastics as introduced by Urbantschitsch, but one can scarcely hope that his plan will rival the oral method owing to its less universal application. One thing is apparent, namely, that "finger-spelling" or the "manual alphabet," and the "sign language" or gestural method, are both disappearing before the more modern modes of teaching mentioned above. The great argument for an oral method is that the deaf person needs some means of communication with the ordinary run of people. The general public do not know the sign language or the manual alphabet, and never will, hence the orally taught have a great advantage over their less fortunate comrades who cannot speak, and depend upon the special education of normally-hearing people, in order to be able to communicate with them other than by writing. On paying a visit to a school for the oral teaching of the deaf, the pupils can be seen in all stages of their progress, "from the little ones just lisping their first words, to those in the higher classes struggling with problems in algebra and the political history of our country." In conversation, it is difficult to understand some pupils at first, just as they, being unaccustomed to the peculiarities of the visitor, are not at first able to read his speech, seeing that no two people speak alike. Another reason is that a stranger talking to a deaf person thinks it necessary to exaggerate his speech, to facilitate his being understood. This is a mistake, and only adds to the difficulty of the lip-reader. The only thing that really helps is to pause slightly between the words but not between the syllables. Three great things have to be accomplished in the education of the deaf—the teaching of language, the teaching of speech, and the teaching of lip-reading. These stand in the order of their importance and difficulty. The speech-training of deaf children should be begun at the same time that a hearing child begins to try to talk, and the deaf child should be treated as nearly as possible as a hearing child. It is the want of observance of this rule and a faulty method, that makes deaf mutes of deaf children, for the latter are perfectly normal in their laughing or crying voice. Hence the importance of the training homes, where infants can be received and early instructed in the correct way. This applies also to the schools, organised on the same scale of equipment as the finest and most expensive establishments for those who can hear. The pupils, being in the enjoyment of all the surroundings of a wealthy and refined home, are carried from the beginning of their instruction through the regular course for a degree in one of the colleges. Nor are these benefits confined to those who have been deaf from infancy. Adults who have become deaf can be taught to understand the conversation of others, and mingle freely in society without their infirmity being even suspected by anyone.

[1] Edin. Med Jnal., Sept., 1895. [2] New York Med. Jnal., June 15, 1895.

